# Ultrasound Evaluation of Transient Ischemic Attack Caused by Styloid Process Elongation: A Case Report

**DOI:** 10.3389/fneur.2019.00026

**Published:** 2019-01-29

**Authors:** Zhaoqiang Li, Yang Hua, Jie Yang, Jingzhi Li

**Affiliations:** Department of Vascular Ultrasonography, Xuanwu Hospital, Capital Medical University, Beijing, China

**Keywords:** styloid process elongation, eagle syndrome, carotid artery, color doppler flow imaging, transcranial doppler

## Abstract

Stylocarotid artery syndrome (SAS) is a type of Eagle syndrome, which is caused by the compression of the internal or external carotid artery (along with their perivascular sympathetic fibers) by an elongated styloid process, resulting in neurological symptoms including transient ischemic attack (TIA) and cervicofacial pain along the distribution of the artery. The authors describe the case of a 56-year-old man with a history of TIA of unknown origin, who underwent color Doppler flow imaging (CDFI) that detected an elongated styloid process and transcranial Doppler (TCD) monitoring that revealed changes in intracranial blood flow when turning his head. To the best of our knowledge, this report is the first to describe the combination of CDFI and TCD to assess changes in extracranial and intracranial blood flow in patients with suspected SAS.

## Introduction

In 1937, Eagle first described the association of cervicofacial pain and other symptoms with an elongated styloid process (1). The syndrome named after him was divided into two types: classic stylocarotid syndrome and stylocarotid artery syndrome (SAS) (2, 3). SAS is essentially a vascular type of Eagle syndrome, which results in neurological symptoms, including transient ischemic attack (TIA) and stroke, due to internal carotid artery (ICA) compression or dissection caused by an elongated styloid process. Three-dimensional (3D) computed tomography angiography (3D-CTA) is the primary imaging modality used to evaluate SAS owing to its ability to clearly depict an elongated styloid process and its adjacent blood vessels (4).

As a non-invasive imaging method, color Doppler flow imaging (CDFI) can depict the bone structure of the neck but also has the advantage of imaging the carotid artery as well as hemodynamics. However, to the best of our knowledge, there are no reports in the literature describing the detection of SAS using CDFI. In this report, we describe a case involving a 56-year-old man with a history of TIA resulting from compression of the right ICA by an elongated styloid process, and explore the important role of CDFI combined with transcranial Doppler (TCD) in the diagnosis of SAS.

## Case Report

A 56-year-old man was admitted to a local hospital for transient (10 min) left limb numbness when he rested on the sofa. Magnetic resonance angiography revealed that the right ICA was occluded from its origin to the intracranial segment. The patient reported taking aspirin, clopidogrel and atorvastatin, and was referred to the authors' center 2 weeks later. Physical examination revealed no obvious positive findings. Although a 2-month history of diabetes was recorded, there was no history of hypertension, hyperlipidemia, or smoking.

**Figure 1:**
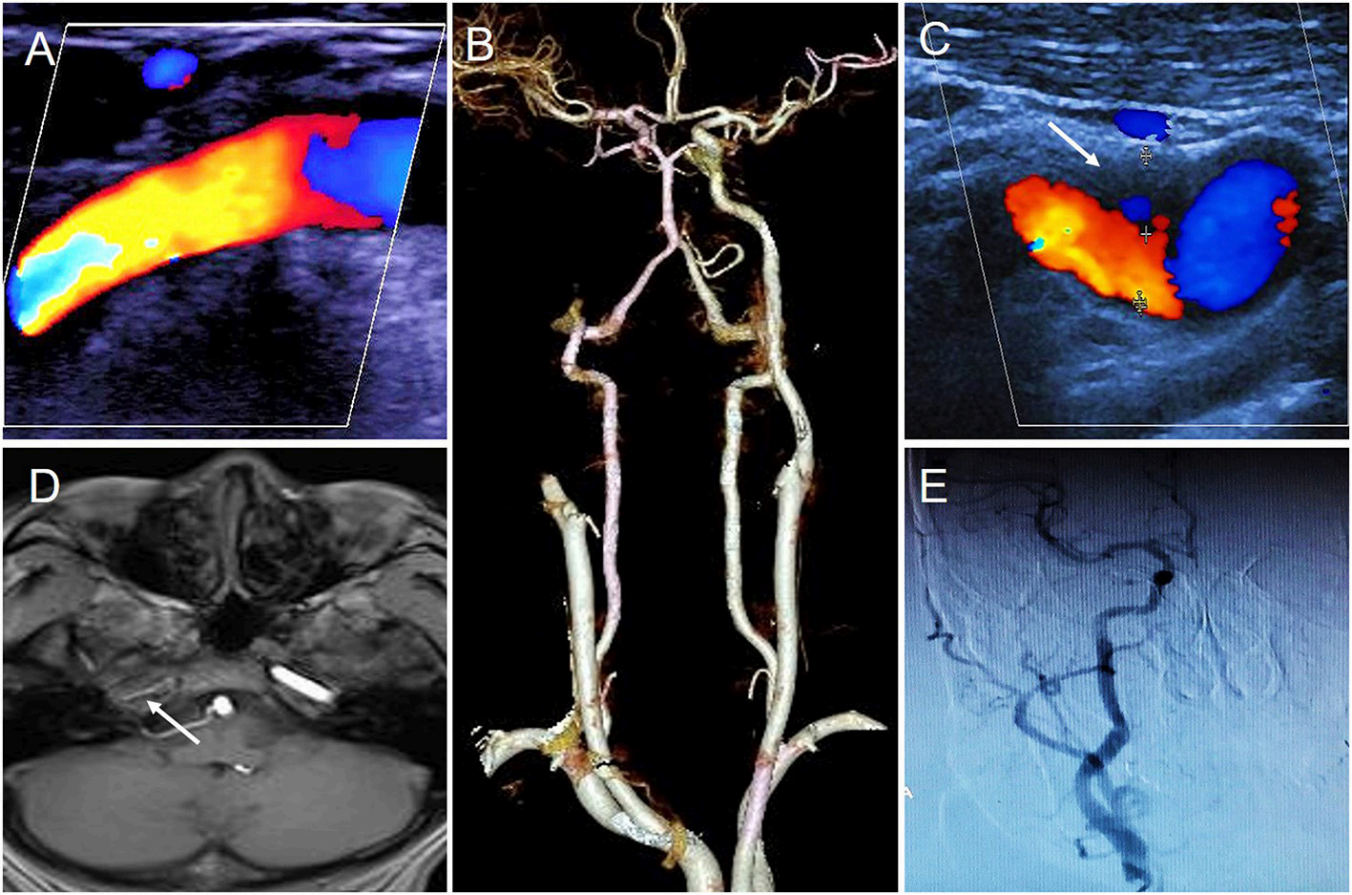
The results of different imaging during the first hospitalization. **(A)** First CDFI showed the right ICA was normal. **(B)** CTA showed the right ICA occluded. **(C)** Second CDFI showed a hypoechoic mass was attached to the anterior wall of initial segment of the right ICA (arrow); **(D)** MRI T1-weighted imageing showed no signal in the intracranial segment of the right ICA (arrow); **(E)** DSA showed right ICA was normal.

The initial CDFI was performed on day 1 of hospitalization, which revealed a patent right ICA with normal blood flow velocity (Figure 1A). However, CTA on day 2 indicated that the right ICA was occluded (Figure 1B). A repeat CDFI scan on day 4 of hospitalization revealed a hypoechoic mass [thickness 2.8 mm (suspected thrombus)] was attached to the anterior wall of the initial segment of the right ICA (Figure 1C). Magnetic resonance imaging (MRI) was performed on day 6; T1-weighted imaging showed that the intracranial segment of the right ICA was invisible, indicating that the right ICA was occluded (Figure 1D). However, digital subtraction angiography (DSA) performed on the same day revealed that the right ICA was normal, with no significant stenosis in any segment (Figure 1E).

CDFI findings were consistent with those of DSA, but nevertheless inconsistent with CTA and MRI. It was not advisable for clinicians to make a definitive diagnosis pertaining to the vascular lesions of the patient. Given the generally good condition of the patient and no significant discomfort, he continued taking aspirin 100 mg/day, clopidogrel 75 mg/day, and atorvastatin 20 mg/day after discharge. Follow-up CDFI 1 and 3 months after discharge did not detect the hypoechoic mass that was attached to the anterior wall of the initial segment of the right ICA, and complete patency and normal blood flow velocity were apparently restored.

**Figure 2:**
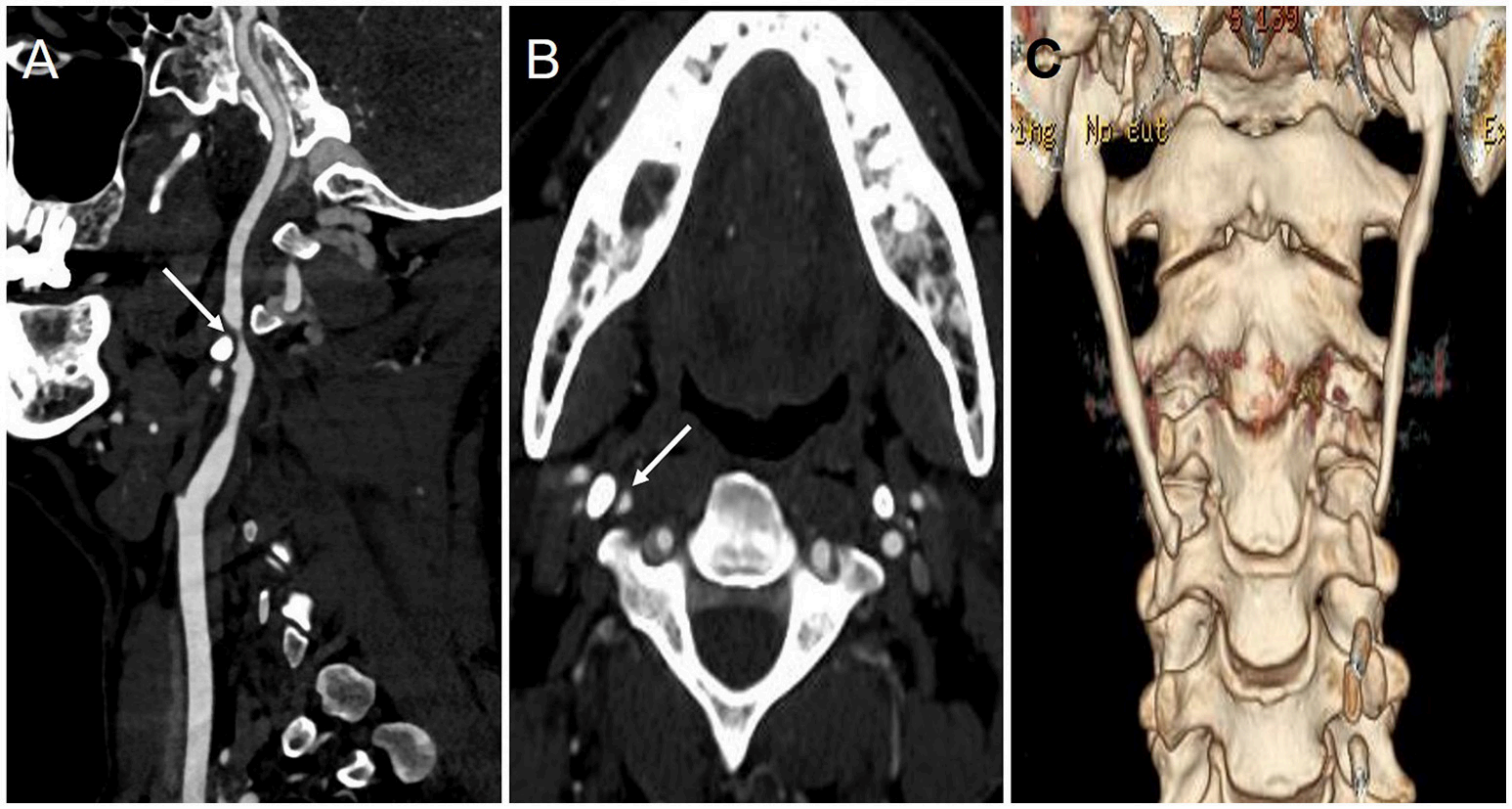
The results of CTA and 3-D CT reconstruction during the second hospitalization. **(A,B)** The sagittal **(A)** and axial **(B)** imaging of CTA showed the right ICA became significantly narrow (arrow) due to styloid process compression; **(C)** 3-D CT reconstruction imaging showed bilateral styloid process were overgrown.

Five months later, however, the patient was re-admitted to the authors' hospital again due to transient (~10 min) left limb weakness without obvious cause. Repeat CTA revealed severe stenosis of the right ICA, and the sagittal image revealed the right styloid process compressing the right ICA horizontally at the C2-3 intervertebral disc (Figures 2A,B). Subsequent 3D computed tomography reconstruction of the cervical spine revealed a bilateral overgrown styloid processes, which was ~6.3 cm in length on the right side and 6.1 cm on the left side (Figure 2C).

**Figure 3:**
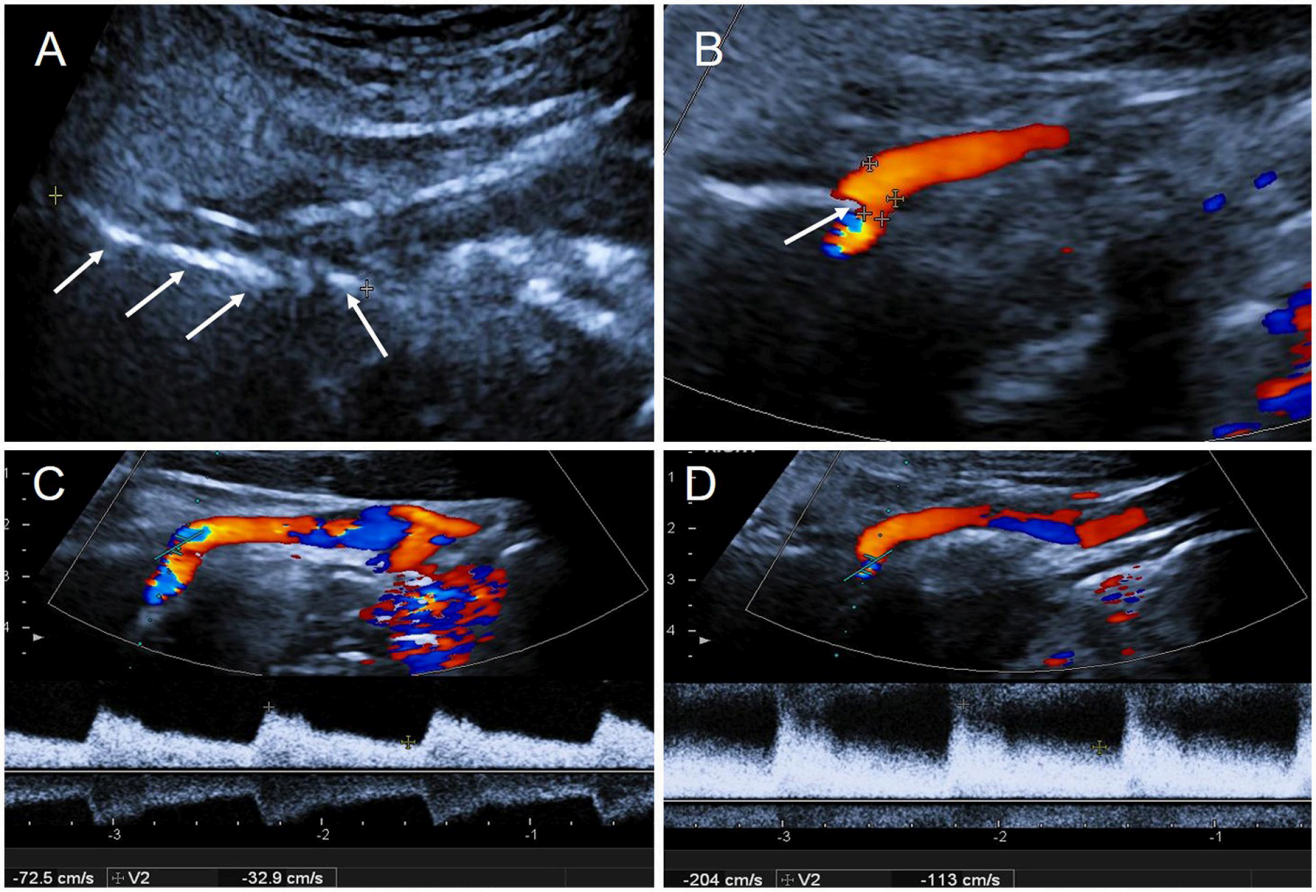
The results of CDFI during the second hospitalization. **(A)** Two-dimensional gray-scale imageing showed a long hyperechoic segment on the right side of neck: styloid process (arrows); **(B)** CDFI showed the right ICA was oppressed by the styloid process (arrow); **(C)** The blood flow velocity in right ICA in normal position: PSV = 72 cm/s, EDV = 33 cm/s; **(D)** The blood flow velocity in right ICA increased when the patient slowly turned head to the right: PSV = 204 cm/s, EDV = 113 cm/s. PSV, peak systolic velocity; EDV, end diastolic velocity.

Subsequently, CDFI depicted a long hyperechoic bony structure on the right side of the neck (Figure 3A), located between the base of skull and the ICA compressing the ipsilateral ICA causing visible artery stenosis (Figure 3B). Blood flow velocity in the ICA dramatically increased when the patient slowly turned his head to the right (Figure 3D), which was significantly altered compared with the normal position (Figure 3C).

**Figure 4:**
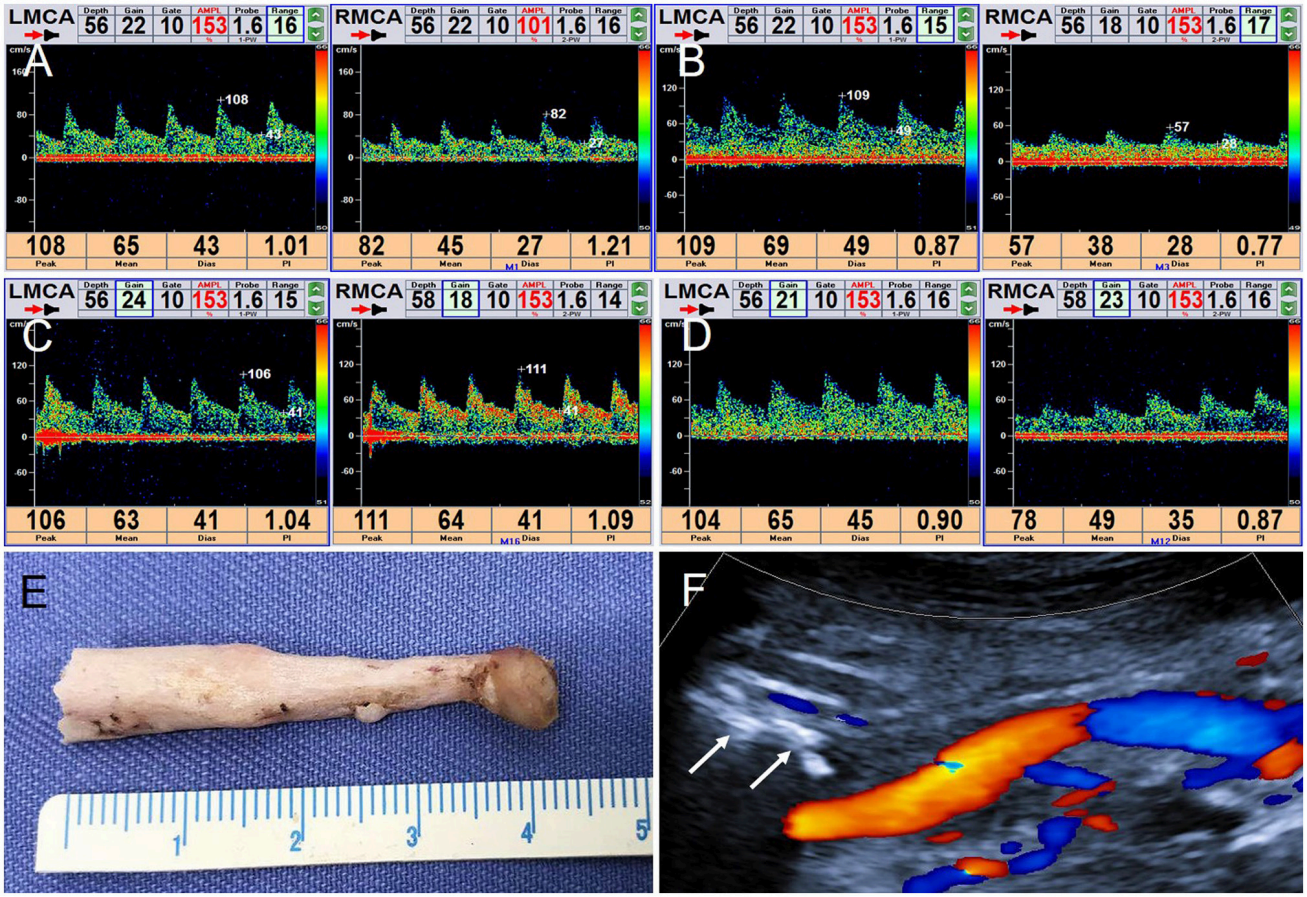
TCD real-time monitoring of blood flow changes in bilateral MCA and postoperative CDFI. **(A)** In normal position, RMCA: PSV = 8 2 cm/s, EDV = 27 cm/s; LMCA: PSV = 108 cm/s, EDV = 43 cm/s. **(B)** Turning his head to the right, RMCA: PSV = 57 cm/s, EDV = 28 cm/s; LMCA: PSV = 109 cm/s, EDV = 49 cm/s. **(C)** Turning his head backwards, RMCA: PSV = 111 cm/s, EDV = 41 cm/s. LMCA: PSV = 1 06 cm/s, EDV = 41 cm/s. **(D)** When the patient turned his head slowly continuously, a visible change of the right MCA and no significant change on the left. **(E)** The right styloid process specimen. **(F)** CDFI detected the styloid process stump (arrow).

TCD was then used to monitor hemodynamic changes of the bilateral middle cerebral artery (MCA) in real time. Even when the patient was in a normal position, blood flow velocity in the right MCA was lower than in the left, but, nevertheless, remained in the normal range (Figure 4A). When the patient turned his head to the right, blood flow velocity in right MCA was significantly decreased (Figure 4B), but increased significantly when he turned his head back to the starting position (Figure 4C). Blood flow velocity in the left MCA did not change markedly during these maneuvers (Figures 4A–C). When the patient turned his head slowly and continuously, blood flow velocity in the right MCA changed dramatically, with no significant change on the left side (Figure 4D).

The patient underwent right styloidectomy. During the operation, the elongated styloid process could be visualized on the front of the right ICA. The middle and lower part of the overgrown styloid process was removed; the length of the specimen was approximately 4.2 cm (Figure 4E). Postoperatively, the right styloid process stump was detected and patency in the right ICA was restored (Figure 4F). TCD was repeated to monitor bilateral MCA in real time; there was no significant change on either side when the patient's head was rotated.

## Discussion

The styloid process is a slender and long bony structure originating from the base of temporal bone and extending anteriorly and inferiorly, whose distal end is attached to three muscles (the styloglossus, stylopharyngeus, and stylohyoid) and two ligaments (the stylohyoid and stylomandibular), with a length ranging from 1.5 to 6 cm (average, 2–3.2 cm) (5). According to previous reports (6, 7), lengths exceeding 3 cm have been defined as an elongated styloid process. Eagle reviewed case reports and estimated the incidence of elongated styloid process to be ~4% (3). However, in an X-ray study, Correll et al. reported an incidence as high as 18% (8), although only a few of these patients developed clinically relevant symptoms (6).

In 1937, Eagle reported that elongation or calcification of the attachment ligament of the styloid process can lead to a series of clinical symptoms, otherwise known as styloid syndrome (also known as “Eagle syndrome”) (1). In his subsequent studies, Eagle classified the styloid syndrome into two types according to the symptoms (2, 3). The classic stylocarotid syndrome is more common, in which patients experience facial or neck pain due to an elongated styloid process compressing nearby cranial nerves, which usually manifests after head rotation and/or neck compression (5). SAS is a relatively rare condition, in which the carotid arteries are compressed by the overgrown styloid process, stimulating the sympathetic nerve fibers around the blood vessels, thus causing pain. In addition, neurological symptoms, such as TIA and stroke, can occur with specific postures or neck movements in SAS patients because the elongated styloid process compresses the ICA causing stenosis or occlusion or even leads to ICA dissection (5).

Zuber et al. first reported a case of ICA dissection caused by an elongated styloid process in 1999 (9). In subsequent reports (10–12), dissection was diagnosed using DSA and MRI or CDFI, and the close positional relationship between the elongated styloid process and the ICA was revealed by 3D-CTA. To our knowledge, Chuang et al. reported the first case of ICA compression caused by an elongated styloid process, which was confirmed by CTA (13). In 2009, Farhat et al. first applied DSA to confirm the compression of an elongated styloid process on the ICA, which led to cerebral ischemia as a result (14). In other reports (15–17), patients with SAS developed TIA or other cerebral ischemic symptoms in specific positions or maneuvers, such as neck rotation, and 3D-CTA confirmed compression of the ICA by an elongated styloid process.

The patient in our case was similar to the patients described in previous reports, who developed TIA due to an overgrown styloid process compressing the right ICA, causing severe stenosis or even transient occlusion. Consequently, perfusion of the right cerebral hemisphere decreased, which led to TIA in the left limb. Several studies have suggested that styloidectomy is an effective treatment for SAS (18, 19). The patient in this case underwent styloidectomy, and TIA did not recur after surgery.

The finding of a hypoechoic mass on the anterior wall of the initiating segment of the right ICA during the patient's second CDFI was considered a mural thrombus. However, the thrombus did not lead to artery occlusion, which was inconsistent with the results of CTA. This may be because the right ICA was severely narrowed or even obstructed, resulting from compression by the styloid process when the patient underwent CTA in the supine position. As a result, CTA enhancement media could not enter the ICA lumen, which obscured visibility of the right ICA. Meanwhile, intra-arterial thrombus can result from prolonged blood flow stagnation. Unlike the position in CTA, the patient's head is turned slightly left when CDFI is used to examine the right ICA; consequently, artery patency is restored through alleviation of compression of the right ICA by the elongated styloid process. After detecting the right elongated styloid process using 3-D CT reconstruction during the second hospitalization in this case, CDFI was used to examine the bony structure of the neck, and a long hyperechoic mass, which protruded into the right ICA, was explored. When the patient slowly turned his head to the right, the increasing velocity of the right ICA blood flow confirmed that the right ICA was compressed. Previous studies describing the evaluation of SAS using CDFI reported a lack of positive signs (13). In the present case, TCD was used to monitor MCA blood flow in real time, and changes in blood flow in the right MCA during rotation of the patient's neck were observed dynamically. Blood flow velocity in the right MCA significantly decreased when the patient turned his head to the right, which explained the clinical symptoms associated with cerebral ischemia. Postoperative TCD monitoring of blood flow in the MCA was not affected by changes in body position, further confirming that intracranial blood flow returned to normal after relieving compression of the right ICA by the elongated styloid process. Therefore, the combination of CDFI and TCD to assess intracranial and extracranial blood flow provides a hemodynamic basis for the correct diagnosis of SAS.

## Conclusion

The combination of CDFI and TCD provides an important, objective, and non-invasive method for detecting an elongated styloid process and assessing changes in the ICA and intracranial blood flow in patients with clinically suspected SAS.

## Ethics Statement

All clinical data in this case report were either provided by the patient or collected by our team's members with the consent of him. There was no additional invasive test or experimental drugs used out of order for the patient. The study was approved by the institutional review board of Xuanwu Hospital, Capital Medical University. The patient gave his written informed consent to participate in the study and to publication of this case report.

## Author Contributions

ZL followed up the patient and compiled the case data and drafted the manuscript. YH helped to design the manuscript and contributed with critical review. JY and JL participated in the modification of the manuscript. All authors contributed to manuscript revision, read, and approved the submitted version.

### Conflict of Interest Statement

The authors declare that the research was conducted in the absence of any commercial or financial relationships that could be construed as a potential conflict of interest.
